# Development of Polypyrrole Modified Screen-Printed Carbon Electrode Based Sensors for Determination of L-Tyrosine in Pharmaceutical Products

**DOI:** 10.3390/ijms22147528

**Published:** 2021-07-14

**Authors:** Ancuța Dinu, Constantin Apetrei

**Affiliations:** Faculty of Science and Environment, “Dunărea de Jos” University of Galati, 800008 Galati, Romania; ancuta.dinu@ugal.ro

**Keywords:** L-Tyrosine, polypyrrole, sensor, amino acid, cyclic voltammetry, chronoamperometry

## Abstract

Good health, of vital importance in order to carry out our daily routine, consists of both physical and mental health. Tyrosine (Tyr) deficiency as well as its excess are issues that can affect mental health and can generate disorders such as depression, anxiety, or stress. Tyr is the amino acid (AA) responsible for maintaining good mental health, and for this reason, the present research presents the development of new electrochemical sensors modified with polypyrrole (PPy) doped with different doping agents such as potassium hexacyanoferrate (II) (FeCN), sodium nitroprusside (NP), and sodium dodecyl sulfate (SDS) for a selective and sensitive detection of Tyr. The development of the sensors was carried out by chronoamperometry (CA) and the electrochemical characterization was carried out by cyclic voltammetry (CV). The detection limits (LOD) obtained with each modified sensor were 8.2 × 10^−8^ M in the case of PPy /FeCN-SPCE, 4.3 × 10^−7^ M in the case of PPy/NP-SPCE, and of 3.51 × 10^−7^ M in the case of PPy/SDS-SPCE, thus demonstrating a good sensitivity of these sensors detecting L-Tyr. The validation of sensors was carried out through quantification of L-Tyr from three pharmaceutical products by the standard addition method with recoveries in the range 99.92–103.97%. Thus, the sensors present adequate selectivity and can be used in the pharmaceutical and medical fields.

## 1. Introduction

We live in times with a lot of restrictive measures meant to stop the spread of severe acute respiratory syndrome-coronavirus (SARS-CoV-2), and numerous papers published by experts in psychology as well as medicine draw attention to the fact that the isolation and quarantine have affected everyone and have led to an increase in the number of depression, emotional disorders, anxiety, and sleep issues [1,2,3]. The negative effects that affect the health of the individual caused by the new coronavirus can be partially or completely treated with pharmaceuticals containing a compound, which acts on the central nervous system, namely tyrosine (Tyr), the target compound of this study. Tyr is a chemical compound discovered in 1846 by German chemist Justus von Liebig, an important compound for a healthy nervous system as it is the precursor of the main neurotransmitters, respectively dopamine, adrenaline, and noradrenaline [4,5]. Low levels of Tyr in human bodies can lead to diseases such as albinism and alkaptonuria whereas high levels of Tyr can lead to different emotional disorders, fits of depression, and even Parkinson’s disease [6,7,8]. On the other hand, Tyr is also an important AA for herbivores, being a basic element of proteins [9,10].

**Figure 1:**
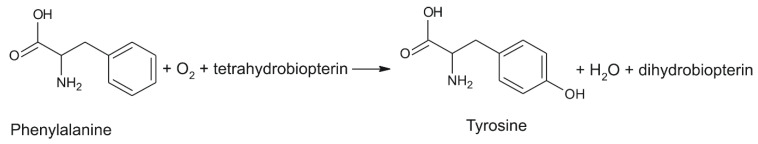
Conversion of Phe in Tyr (adapted from [16]).

For the human body, Tyr is a non-essential AA synthesized in the liver through the conversion of the essential AA phenylalanine (Phe), according to Figure 1, but it can also be assimilated through external sources by including in the diet foods such as dairy products, meat, fish, eggs, walnuts, beans, oatmeal, and wheat [11,12]. In addition, Tyr is a compound that can be found in many pharmaceutical supplements aimed to help with inborn disorders of metabolism known as phenylketonuria (PKU) [13,14,15].

On the other hand, Tyr, together with zinc, magnesium, manganese, and iodine helps to improve the role of the thyroid gland, reducing disorders such as ponderal gain, edema, muscular spasms as well as brittle hair and nails [17,18].

In the literature, there are a number of methods used to determine Tyr in both human fluids and pharmaceutical products, and in this research study is presented the development of novel sensors for a faster, easier, and sensitive detection of this AA [12,19,20,21,22]. Many of the classical methods used to detect Tyr are sensitive and selective but require expensive equipment and a difficult and long processing technique such as liquid chromatography-mass spectrometry (LC-MS) [23], high-performance liquid chromatography (HPLC) with ultraviolet (UV) detection [24], chemiluminescence [25], fluorimetry [26], and capillary electrophoresis [27].

However, in recent years, faster and cheaper detection methods have been developed with high precision and sensitivity based on electroanalytical methods using potentiometric [28], optical [29], colorimetric [30], or voltamperometric sensors [31,32,33].

The usual voltamperometric methods used for L-Tyr detection reported in the literature are differential pulse voltammetry [34], cyclic voltammetry [35,36], chronoamperometry [37], linear sweep voltammetry [38,39], and square wave voltammetry [40].

Different sensitive materials could be used for the development of sensors such as PPy, a polymer from the conducting polymers (CPs) class, an important field of research of interest, as in 2000, the Nobel Prize for Chemistry was awarded to three researchers with outstanding results in this field [41]. The synthesis and properties of PPy have been studied since 1916 when hydrogen peroxide was used for the chemical oxidation of pyrrole. PPy was obtained in the form of “black pyrrole” [30,31]. PPy synthesis can also be carried out electrochemically by means of CA, chronopotentiometry, and CV [42,43,44].

The various practical applicabilities of PPy are due to its numerous useful properties, the most important being its malleability, stability, electrical conductivity, and biocompatibility. These properties make it useful in different fields such as biology, medicine, pharmacy, and chemistry [44]. PPy is one of the most widely used conducting polymers in developing electrochemical sensors and biosensors for different analytes of interest. Regarding PPy usefulness in sensor development, being a conjugated polymer positively charged and doped with different anionic species, it brings a series of benefits such as high current density, fast electron transfer, biocompatibility, easy preparation, air stability, and its capacity to integrate a large variety of ion doping [45].

Following research on PPy and its use in different applicative fields, it was found that certain thermic (stability), electrical (conductivity), and morphological (permeability) PPy properties need improvement [42]. Thus, for PPy chemical synthesis, many researchers have used different oxidants such as ferric chloride (FeCl_3_) or ammonium persulfate. Electrosynthesis and doping with chloride (Cl^−^), sulfate (SO_4_^2−^), tetrafluoroborate (BF_4_^−^), and perchlorate (ClO_4_^−^) have also been employed. Few studies have reported PPy doped with sodium dodecyl sulfate (SDS), potassium hexacyanoferrate (II) (FeCN), sodium Nitroprusside (NP), dodecylbenzene sulfonate (DBS), and p-toluene sulfonate (PTS) in different organic solvents or in aqueous environments, which could lead to high performances, respectively high conductivity, high thermic stability, reproducibility, etc. [34,35]. One advantage of electrochemical synthesis is the possibility of carrying out the deposition on the electrode and the doping of the polymer in one stage, a procedure useful in the case of electrochemical sensor development [46].

In this research regarding L-Tyr detection, new sensors were developed through the modification of screen-printed carbon electrodes with PPy doped with electroactive and surfactant compounds by the CA method. In the case of electropolymerization, an adequate potential has been applied in order to induct the oxidation reaction in the presence of a doping agent, which can ensure the electroneutrality of the polymeric film [47,48,49]. All these reasons together determined and led to research regarding the development, characterization, and use of new voltamperometric sensors based on PPy modified screen-printed electrodes, doped with three doping agents in order to detect and quantify with increased sensitivity, selectivity, and reproducibility of the AA L-Tyr present in pharmaceutical products, respectively: PPy/FeCN-SPCE, PPy/DSA-SPCE, and PPy/NP-SPCE. This study is important because such sensors modified with PPy doped with electroactive compounds for the detection of L-tyrosine in pharmaceuticals by electrochemical methods have not been developed so far. The novelty is related to development of sensors for the detection of L-Tyr in complex pharmaceutical samples with appropriate sensitivity and selectivity. Complex pharmaceutical samples are difficult samples to analyze due to a huge number of components and possible interference among these. The analysis of such samples was successful accomplished in this research. The sensor PPy/FeCN-SPCE was validated at the laboratory level and it could be used in routine analysis for the quality control of pharmaceutics.

## 2. Materials and Methods

### 2.1. Chemicals and Solutions

L-Tyr (≥98%), potassium chloride (≥99.0%) (KCl), FeCN (≥99.5%), SDS (≥99.0%), NP (≥99%), pyrrole (98%), L-tryptophan, L-cysteine, and L-phenylalanine were purchased from Sigma-Aldrich (St. Louis, MO, USA).

The preparation of the solutions of these compounds was performed with ultrapure water (18.3 MΩ × cm, Milli–Q Simplicity^®^ Water Purification System from Millipore Corporation, Burlington, MA, USA).

### 2.2. Equipment

The electrode modifications with PPy by the CA method were carried out using the EG&G potentiostat/galvanostat (Princeton Applied Research, Oak Ridge, TN, USA), 263A model, controlled by ECHEM software. The SPCEs were purchased from Metrohm-Dropsens (www.dropsens.com) (accessed on 22 April 2021). An Ag/AgCl/KCl_3M_ electrode was used as the reference electrode, the counter electrode was a Pt wire, and the working electrode was a screen-printed carbon electrode.

A Biologic SP 150 potentiostat/galvanostat (Bio-Logic Science Instruments SAS, Seyssinet-Pariset, France) was used for the electrochemical measurements and the software used for control and data acquisition was EC–Lab Express. The electrochemical cell used for the analysis of the samples had a capacity of 15 mL and the reference electrode and the counter electrode were those integrated in the commercial sensor device (counter electrode–carbon, Ag/AgCl reference electrode). For the analysis of the experimentally obtained voltammograms, Microsoft Excel software was used.

A JEOL T-300 scanning electron microscope was used for the surface morphology studies. For dissolving and homogenization of all solutions, an Elmasonic S10H ultrasonic bath was used.

### 2.3. Sensor Configuration and Methodology

For the detection of L-Tyr, screen-printed carbon electrodes purchased from Metrohm-Dropsens (SPCE—model DRP- C110, diameter of 4 mm, surface of 12.56 mm^2^) were modified with PPy. For PPy deposition, three different solutions of monomer/doping agent (FeCN, SDS and NP) 0.1 M/0.1 M were used. An ultrasonic bath was used in order to homogenize the solutions by ultrasonication for 5 min. For SPCE modification, the CA method was used by applying a potential 0.8 V during 90 s at a constant temperature of 25 °C. After being manufactured, the sensors were rinsed with ultrapure water and kept in the dark at 4 °C until use. By modification of the SPCE surface, the following chemically modified sensors were obtained: PPy/FeCN-SPCE, PPy/SDS-SPCE, and PPy/NP-SPCE.

Then, the electrochemical behavior of PPy sensors PPy/FeCN-SPCE, PPy/SDS-SPCE, and PPy/NP-SPCE was analyzed through the CV technique in a solution of 0.1 M of KCl and then in a double solution containing 10^−3^ M L-Tyr and 0.1 M KCl. In the case of CV, the established parameters were: initial potential 0.0 V, positive vertex potential 0.5 V, negative vertex potential −1.0 V, and the scan rate was between 0.1 and 1.0 V × s^−1^. For the CV studies with the polypyrrole based sensors immersed in KCl and KCl + Tyr solutions, the screen-printed reference electrode Ag/AgCl was used. The counter electrode was a screen-printed C electrode integrated on the commercial electrode DRP-110. Six cycles were recorded in the solution to be analyzed in order to stabilize the sensor signals. All the results reported in this study are after the stabilization stage. Prior to the electrochemical measurements, the electrolyte solution was deoxygenated with nitrogen.

### 2.4. Samples Tested

In order to validate the results obtained with modified electrodes, we selected three pharmaceutical products containing L-Tyr in different concentrations, from three different producers, one of the products containing more AAs. Thus, we analyzed L-Tyrosine (SOLARAY) (500 mg L-tyrosine), Tiroidin (PARAPHARM) (90 mg tyrosine), and Cebrium (EVER NEURO PHARMA) (4.012 mg L-Tyrosine).

Compared to L-Tyrosine 500 mg SOLARAY, which is a product that contains only the amino acid under study, the other two pharmaceuticals also contain other active compounds, in addition to the amino acid L-tyrosine, as follows: Tiroidin PARAPHARM–spirulina, vitamin E (alfa-tocoferol), iodine (in the form of potassium iodine), selenium (in the form of sodium selenite), and Cebrium EVER NEURO PHARMA—glutamic acid, lysine, leucine, arginine, aspartic acid, serine, phenylalanine, valine, threonine, tyrosine, isoleucine, histidine, methionine, and tryptophan.

For the electroanalysis of the pharmaceutical products, these were triturated to obtain a homogenous powder. A part of this powder was weighed and dissolved in 0.1 M KCl solution and ultra-sonicated for 15 min. The unsolved excipients were removed from the solution by filtration. Knowing the amount of L-Tyr in the pharmaceutical products, the amount of solid powder dissolved, and the volume of KCl solution added, the concentration of L-Tyr was calculated. These solutions, prepared from pharmaceutical products, were used for the validation of the sensor by the standard addition method.

## 3. Results and Discussions

### 3.1. Deposition of the PPy on the Surface of SPCEs by Chronoamperometry

PPy, a CP that offers sensors higher sensitive properties, as previously mentioned, was used to modify SPCEs, the electrosynthesis being achieved in the presence of three doping agents.

**Figure 2:**
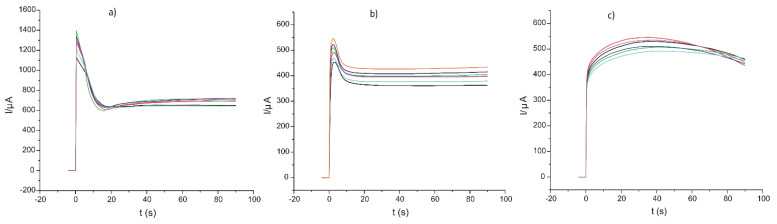
Current versus time curves registered during the electrosynthesis of six different sensors prepared in the same conditions (**a**) PPy /FeCN, (**b**) PPy/NP, and (**c**) PPy/SDS.

The doping agent/monomer solutions were each introduced into the electrochemical cell, the three electrodes were connected, and a potential of 0.8 V was applied to the working electrode during 90 s. The chronoamperograms obtained for six different sensors developed from the same solution are presented in Figure 2.

As can be observed in Figure 2, the process of electrodeposition is strongly influenced by the physical and chemical properties of the doping agent. The dynamic of the processes was different, the deposition rate being higher in the case of FeCN.

**Figure 3:**
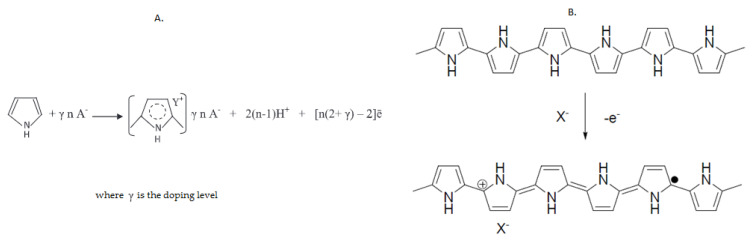
(**A**) General reaction of pyrrole electropolymerization (Reprinted from [50] with permission of the publisher); (**B**) Molecular structure of PPy and general doping process [51].

The general PPy electrosynthesis [50] and doping process [51] are shown in Figure 3A,B, respectively.

The doping agents used in this research were selected according to a series of characteristics: electroactivity, multiple charges, and large molecular weight. These characteristics should improve the stability of the sensitive layer as well as the sensitivity and selectivity.

**Figure 4:**
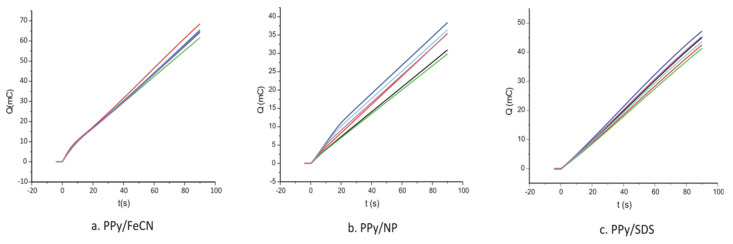
Q versus time (t) curves registered during electrosynthesis of six different sensors prepared in the same conditions in the presence of (**a**) FeCN, (**b**) NP, and (**c**) SDS.

In order to determine the thickness of the polymer layer deposited on the surface of the electrode, the chronoamperograms were presented in the form of electrical charge (Q) as function of time (Figure 4).

Figure 2 and Figure 4 show the chronoamperometric curves corresponding to six sensors prepared from the same solution pyrrole/doping agent in order to verify the reproducibility of the preparation method. As can be seen, the differences between the sensors prepared under the same conditions were very small, the coefficient of variation being less than 5%.

From the electrochemical charges employed for each electropolymerization, knowing the surface of the electrode (A/cm^2^), the thickness of the polymer layer deposited on the electrode was calculated, according to Equation (1):

(1) $$d=\frac{{\mathrm{QM}}_{w}}{\mathrm{zFA}\rho }$$

where Q is the oxidation charge; M_w_ is the molecular mass of the monomer (M = 67.0892 g/mol); z is the number of electrons; F is the Faraday constant; A is the surface area of the electrode; and ρ is the polymer density ($\rho_{\mathrm{PPy}}=1.5\;g/{\mathrm{cm}}^{3}$) [52]. The values obtained are presented in Table 1.

As it can be seen from the table, the thickness of the polymer film largely depends on the nature of the doping system and its redox properties. The thickness of the films, in the nanometric range, is optimal for a sensitive detection of L-Tyr. It should be said that thicker PPy films can be achieved in the case of FeCN because this doping agent is more redox active and the electrodeposition kinetics are faster compared to the other doping agents.

**Figure 5:**
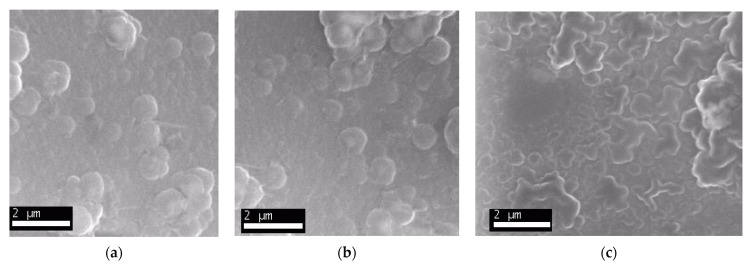
Scanning electron microscopy images of the sensitive element of polypyrrole based sensors doped with (**a**) FeCN, (**b**) NP, and (**c**) SDS.

The morphology of the polymeric films was studied by scanning electron microscopy and the images obtained are presented in Figure 5.

The polypyrrole films doped with different doping agents showed different morphologies, the polymer being formed mainly from spheres of various sizes. These differences are related to the nature of the doping agent and the different deposition rate of the PPy film.

### 3.2. Voltammetric Responses

#### 3.2.1. Electrochemical Responses of Sensor Immersed in 0.1 M KCl Solution and in 0.1 M KCl–10^−3^ M L-Tyr Solution before Modification

**Figure 6:**
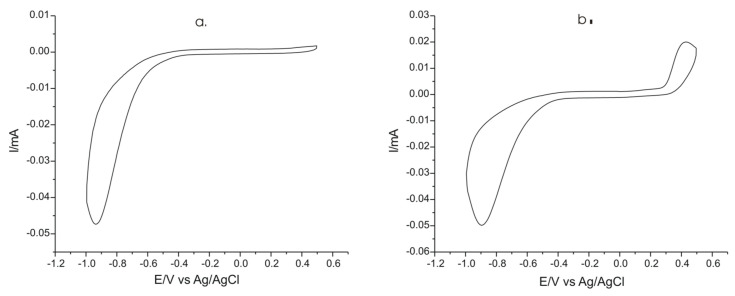
Electrochemical response of carbon screen-printed electrode immersed in: (**a**) 0.1 M KCl and (**b**) 0.1 M KCl and 10^−3^ M L-Tyr solution at 0.1 V × s^−1^.

The electrochemical response of unmodified carbon screen-printed electrodes was investigated in two solutions: 0.1 M KCl solution, respectively in 0.1 M KCl–10^−3^ M L-Tyr solution (Figure 6) in the potential range from 1.0 to 0.5 V.

This step is important to be able to compare the results obtained with the unmodified sensor with modified electrodes with PPy doped with FeCN, N,P and SDS. As observed in cyclic voltammograms presented in Figure 6, the unmodified electrode immersed in both solutions have a potential window ranging from approximately −0.5 to +0.5 V. For the improvement of the electrochemical properties of the electrode for Tyr detection, modification with a sensitive and selective material is necessary.

#### 3.2.2. Stable Electrochemical Responses of Sensors in 0.1 M KCl Solutions and in Double Solution of 0.1 M KCl–10^−3^ M L-Tyr Solutions

The electrochemical behavior of sensors was initially studied in a 0.1 M KCl solution, as this solution favors observing redox processes of the modifying material on the electrode surface, often being used to characterize chemically modified sensors, for example, CPs doped with different anions. The optimal potential range used was from −1.0 V to +0.5 V, and the scan rate was 0.1 V × s^−1^.

After preparation, the sensors were introduced in the 0.1 M KCl solution and the cyclic voltammograms were recorded. The first three cycles were somehow different compared with the subsequent ones, and this is related to the stabilization of the PPy layer in the electrolyte solution. After six cycles, the sensor responses became stable [53].

**Figure 7:**
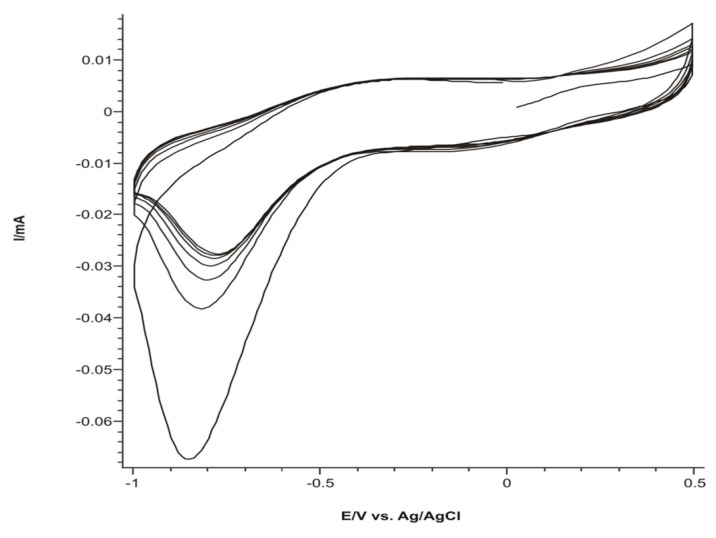
Six successive cyclic voltammograms of the PPy/FeCN sensor in 0.1 M KCl solution.

An example of six repetitive cyclic voltammetric scans is presented in Figure 7.

**Figure 8:**
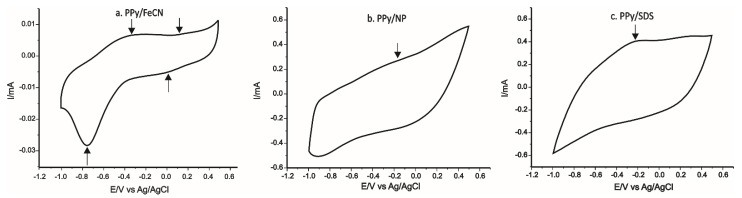
Stable response of polypyrrole based sensors immersed in 0.1 M KCl solution at 0.1 V × s^−1^: (**a**) PPy /FeCN-SPCE; (**b**) PPy/NP-SPCE, and (**c**) PPy/SDS-SPCE.

The stable responses of the sensors in the 0.1 M KCl solution are presented in Figure 8.

In the case of PPy/FeCN-SPCE, we noted two anodic peaks and two cathodic peaks, which corresponded, on one hand, to the PPy redox process, whereas the II redox process corresponded to the oxidation–reduction process of potassium ferrocyanide found in the polymeric matrix. In the case of PPy/NP-SPCE and PPy/SDS-SPCE, the peaks were less defined because of the high background current and because SDS is not electroactive. Peak currents and potential achieved from CVs for each sensor are collected in Table 2.

It can be noted that all peak characteristics (potential, intensity, form) achieved through the KCl 0.1 M solution were related to the chemical nature of doping compounds included in the polymeric matrix [46]. It can be expected that a higher sensitivity can be achieved in the case of PPy/FeCN-SPCE because the peaks recorded at PPy/FeCN-SPCE in 0.1 M KCl were better defined compared with the other sensors (higher ratio between the peak and background current).

The peaks achieved have, on one hand, characteristics belonging to the PPy chain redox process and, on the other hand, they reflect the doping activity of the redox in the polymeric matrix (in the case of FeCN).

The sensor PPy/FeCN-SPCE had better defined peaks and a relative reduced background current opposed to the other two sensors.

**Figure 9:**
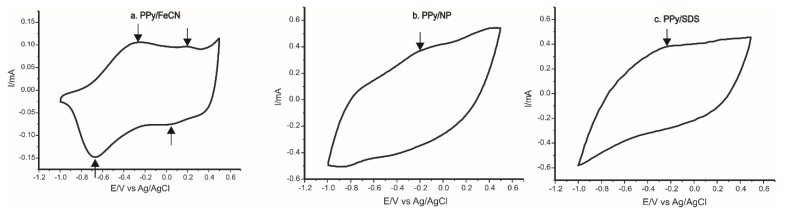
Stable response of polypyrrole based sensors immersed in 0.1 M KCl and 10^−3^ M L-Tyr solution at 0.1 V × s^−1^: (**a**) PPy/FeCN-SPCE, (**b**) PPy/NP-SPCE, and (**c**) PPy/SDS-SPCE.

In the following step, the modified sensors were immersed into a double solution containing 0.1 M KCl–10^−3^ M L-Tyr. On detection of AA L-Tyr, the stable electrochemical responses achieved with the three PPy modified electrodes are presented in Figure 9.

The intensity and potential of anodic and cathodic peaks observed in the cyclic voltammograms are presented in Table 3.

For the electrode PPy/FeCN (Figure 9a) immersed into the 0.1 M KCl and 10^−3^ M L-Tyr solution, two anodic and cathodic peaks were identified, the first redox process appeared at E_paI_ = −0.28 V (peak I) and corresponded to the redox properties of PPy, and the second peak at E_paII_ = 0.19 V corresponded to the redox reaction of the potassium ferrocyanide included in the polymeric matrix [54]. Both peak pairs, related with PPy and FeCN, were influenced by the presence of the L-Tyr by shifting the peak potentials and increasing the peak currents, especially in the anodic scan.

The response of the sensors PPy/NP (Figure 9b) and PPy/SDS (Figure 9c) immersed into a solution of 0.1 M KCl and 10^−3^ M L-Tyr showed a single oxidation–reduction process due to PPy influenced by the nature of the doping agent included in the polymeric matrix.

It can be observed in Table 3 that the values I_pc_/I_pa_ calculated for the three sensors were different, with values close to the ideal one, with the best result being achieved in the case of PPy/FeCN-SPCE. This result proves that the oxidation–reduction processes at the surface of the sensor are quasi-reversible [54].

In the case of PPy/FeCN and PPy/NP, the presence of L-Tyr in the analyzed solution influenced the electrochemical response by shifting the potential of the peaks observed and increasing the peak current by comparing it with the response in the KCl solution, which proves that PPy/FeCN and PPy/NP sensors can be used for L-Tyr detection.

The peaks of PPy/FeCN-SPCE were lower as current in comparison to those observed for the other modified electrodes, but the ratio between the current of the peaks and the background current was better in the case of this sensor. Therefore, the PPy/FeCN-SPCE improved voltammetric responses compared with the other sensors developed in this study and the detection of L-Tyr was more accurate.

#### 3.2.3. The Influence of the Scan Rate on the Responses of the Sensors Immersed into 0.1 M KCl and 0.1 M KCl–10^−3^ M L-Tyr Solutions

It is well known that the scan rate plays an important role in electrochemical measurements as it contributes to bringing out the redox processes and greatly influencing the voltammetric responses of the sensors. The voltammograms were recorded at 10 scan rates, from 0.1 to 1.0 V × s^−1^, in the potential range between −1.0 V and + 0.5 V, thus making possible the study of dynamic characteristics and of sensor signals.

**Figure 10:**
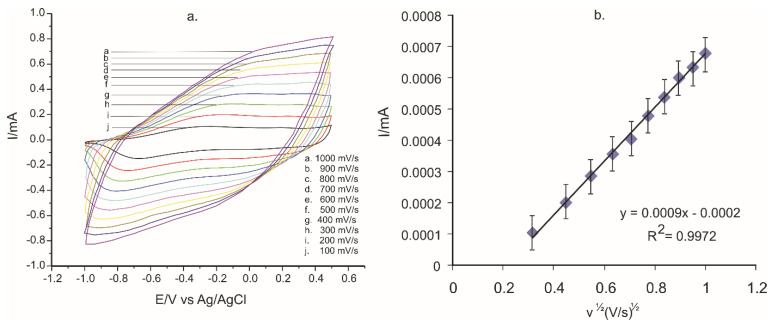
(**a**) CVs of the PPy/FeCN-SPCE sensor immersed in the KCl 0.1 M and 10^−3^ M L-Tyr solution, at scan rates between 0.1 and 1.0 V × s^−1^. (**b**) Linear fitting curves between I_pa_ and square root of scan rate.

Figure 10a presents the CVs obtained with the PPy/FeCN sensor immersed into 0.1 M KCl and 10^−3^ M L-Tyr solutions at scan rates between 0.1 to 1.0 V × s^−1^. It can be noted that there was an increase in peak intensity together with the increase in scan rate. In order to determine the limiting factor of electrochemical reactions, the linear fitting between I_pa_ and the square root of the scan rate for PPy/FeCN was carried out (Figure 10b).

The intensity of peaks is directly proportional to the square root of the scan rates, which points out that the redox processes have a diffusion process to determine the kinetics stage.

The active surfaces of the unmodified and the three modified sensors were calculated from the linear fitting equations by using the Randles–Sevcik equation:

(2) $$I_{p}=\left(2.69\;\times \;10^{5}\right)\;\times \;n^{3/2}\;\times \;A\;\times \;C\;\times \;D^{1/2\;}\;\times \;v^{1/2}$$

where *I_p_* is the peak current (Amperes); n is the number of electrons transferred in the redox process; A is the electrode area (cm^2^); C is the concentration (mol × cm^−3^); D is the diffusion coefficient (cm^2^ × s^−1^); and v is the scan rate (V × s^−1^). The diffusion coefficient of L-Tyr is 3 × 10^−5^ cm^2^ × s^−1^ [55].

The values achieved for A, the active area of the sensor, calculated from the slope of the equation from I_pa_ and v^1/2^ as well as the surface roughness factor are presented in Table 4.

In conclusion, it can be noted that the values of the active area and roughness factor of the PPy/FeCN sensor were the highest, thus this sensor had the highest sensitivity for L-Tyr detection.

#### 3.2.4. Development of Calibration Curve

CV was used to detect L-Tyr in solutions of different concentrations. This is an important stage as the equation of the calibration curve can help calculate the limit of detection (LOD) and the limit of quantification (LOQ) of each PPy-modified sensor.

The concentration range researched was between 0.5–27 × 10^−6^ M for all three electrodes developed in this study. One can note that for all three sensors, there was a linear increase in the anodic intensity peak once the Tyr concentration increased. The sensitivity of the PPy/FeCN-SPCE, PPy/NP-SPCE, PPy/SDS-SPCE, and unmodified SPCE for determination of L-Tyr were: 1.463 A/M, 0.2789 A/M, 0.3412 A/M, and 0.1543 A/M, respectively.

**Figure 11:**
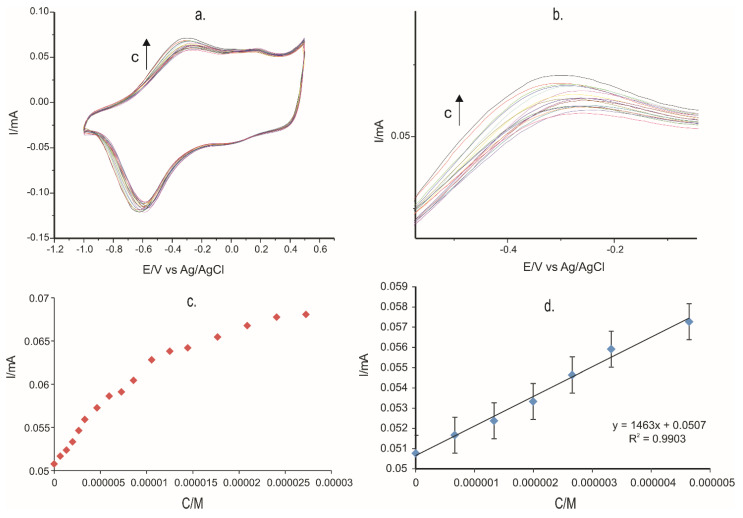
(**a**) Cyclic voltammograms obtained for different concentrations of L-Tyr in the range 0.5–27 × 10^−6^ M. (**b**) The zoom on the anodic peak for which the calibration curve was developed. (**c**) The anodic peak current I variation with Tyr concentration. (**d**) Calibration curve in the range 0.5–5 × 10^−6^ M.

As an example, Figure 11 presents the results obtained with PPy/FeCN-SPCE. Figure 11a shows the electrochemical responses of the PPy/FeCN sensor at different concentrations of L-Tyr in the range 0.5–27 × 10^−6^ M. A zoom of the anodic peaks zone is presented in Figure 11b. In Figure 11c is observed the increment of the anodic peak current I of the sensor PPy/FeCN when the concentration of the L-Tyr solution increased. The dependence is linear when L-Tyr concentrations are lower, followed by a plateau where current increment is not linear, which means that the sensor has reached a saturation level (Figure 11c). In the concentration range 0.5–5 × 10^−6^ M, the current increased directly with the L-Tyr concentration, the dependence having a determination coefficient of 0.99 (Figure 11d).

The voltammetric determinations were achieved with all three sensors, and Table 5 shows the LOD and LOQ obtained through Tyr detection.

The lowest LOD and LOQ values were achieved for the PPy/FeCN-SPCE sensor. The results obtained with the sensors developed in this study are comparable with those obtained with other sensors used for the electrochemical detection of L-Tyr reported in the literature, as presented in Table 6, where some sensors even had lower values of LODs [56,57,58,59,60,61,62,63,64,65]. However, the sensors developed in this study have a series of advantages.

The advantages of polypyrrole based sensors are the simplicity of preparation in a single stage, good reproducibility of the sensors’ preparation, good stability, appropriate sensitivity and selectivity for the detection of tyrosine, low cost, and require low quantities of samples. Furthermore, in the case of PPy/FeCN-SPCE, the presence of an electroactive doping agent in the polypyrrole matrix increases the sensitivity by mediating the electron transfer process.

#### 3.2.5. Precision, Reproducibility, and Stability of the Modified Sensor

Precision studies were performed on L-Tyr solutions of 4 × 10^−6^ M for inter-day and intra-day precision with the PPy/FeCN-SPCE sensor. Intra-day accuracy was achieved at three different times of the day (3 h interval), and inter-day accuracy was evaluated for three different days, and the relative standard deviation (RDS%) values of the voltammetric signals are reported in Table 7.

To evaluate the reproducibility of the sensor modified with PPy and doped with FeCN, measurements were performed for six replicates, using 4 × 10^−6^ M L-Tyr solution. After each experiment, the sensor was taken from the solution, rinsed with ultrapure water, dried in a desiccator, and re-immersed in the solution. The relative standard deviation (RDS) of the six cyclic voltammograms replicates was 3.3%.

Regarding the electrode stability, this was determined in the same L-Tyr solution during three weeks. After three weeks, it was found that the electrode kept 91% of the initial signal response. The sensors were stored in the refrigerator at a temperature of 4 °C in a closed dry box when they were not used.

#### 3.2.6. Interference Study

To evaluate sensor selectivity for L-Tyr detection in complex samples, the influence of several amino acids (tryptophan, cysteine, and phenylalanine) was studied as these amino acids could interfere in electroanalysis. The concentration of the L-Tyr was 4 × 10^−6^ M and the detection technique was cyclic voltammetry. The tolerance limit was calculated as being the maximum concentration of the interfering amino acids, which causes a relative error of ±5% for the tyrosine quantification. The results obtained are shown in Table 8.

The quantification of L-Tyr is influenced by the presence of other AAs in the analyzed solution, the relative errors being close to ±5% and recoveries in the range 95.3–104.575%. Based on these results, it could be concluded that PPy/FeCN-SPCE is selective for L-Tyr quantification in samples containing up to 0.001 M of tryptophan, cysteine, and phenylalanine.

#### 3.2.7. Validation of Modified Sensors on Real Samples

Three pharmaceutical products from three different manufacturers with different Tyr concentrations were selected and analyzed in order to validate the sensors made in this research through AA L-Tyr quantification. The samples were obtained from a local pharmacy. The three pharmaceutical products used were L-tyrosine (Solaray) (L-Tyr 500 mg), Tiroidin (Parapharm) (L-Tyr 90 mg), and Cebrium (Ever Neuro Pharma) (L-Tyr 4.012 mg) and they were analyzed by CV.

The amounts of AAs in the pharmaceutical product Cebrium are 4.012 mg L-Tyr, 1.02 mg tryptophan, and 4.012 mg phenylalanine. Therefore, the concentrations of each AA in the solution analyzed were at the similar level. Taking into account the results obtained in the interference studies, the concentration of the interfering AAs were under the concentration threshold values, which can cause a relative error of ±5% for the tyrosine quantification.

PPy/FeCN-SPCE was used to detect and quantify L-Tyr in the pharmaceutical products as it had the best analytical performances among the three sensors developed in this research study.

**Figure 12:**
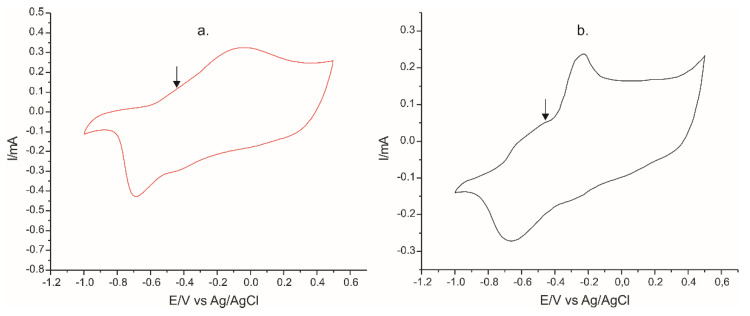
Voltammetric responses of the PPy/FeCN sensor in a solution of: (**a**) L-Tyrosine (SOLARAY); (**b**) Cebrium (EVER NEURO PHARMA). Estimated concentration of L-Tyr in the solutions was 10^−3^ M, in agreement with the quantity declared by producers in the pharmaceutical products.

The cyclic voltammograms recorded with PPy/FeCN-SPCE for the two pharmaceutical products containing 10^−3^ M L-Tyr in the analyzed solution are shown in Figure 12.

The CVs of the sensor immersed in the pharmaceutical sample solutions were somehow different when compared with the CV in the model solutions. The CV in the solution of Cebrium was more complex and different when comparing the CVs in Tyr solution or Solaray solution. This is related with the presence of different amino acids in this sample.

**Figure 13:**
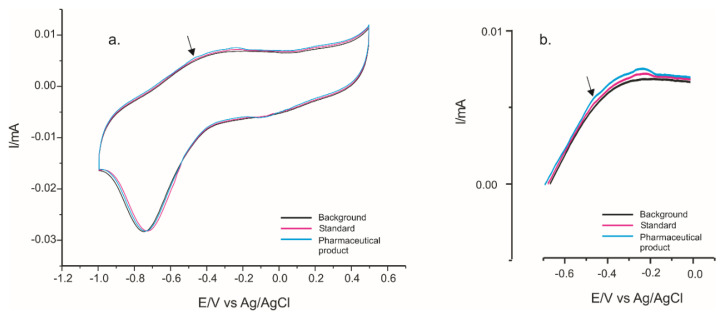
(**a**) Cyclic voltammograms of the PPy/FeCN sensor in solution KCl solution (background), L-Tyr solution (standard spike, 2 × 10^−6^ M), Cebrium spike solution (estimated total concentration 4 × 10^−6^ M of L-Tyr). (**b**) Zoom of the cyclic voltammograms in the zone of L-Tyr detection.

However, the influence of interfering compounds was low when the concentrations of L-Tyr and interfering species were low. For example, in Figure 13a,b, the responses of the PPy/FeCN-SPCE in 0.1 M KCl solution (background), in 2 × 10^−6^ M solution of standard spike (solution obtained from pure L-Tyr), and in pharmaceutical product Cebrium spike solution corresponding to 2 × 10^−6^ M L-Tyr added (estimated L-Tyr total concentration 4 × 10^−6^ M) are presented. The area where the Tyr signal appears is magnified (Figure 13b).

For the quantitative analysis of pharmaceutical products, the standard addition method was used. The spike and recovery experiments were carried out by measuring CV responses for pharmaceutical samples in which a known concentration of L-Tyr was added. Three additions of L-Tyr standard solution were carried out in the solution of each pharmaceutical product. The results obtained are presented in Table 9. The analyses were carried out in triplicate for each pharmaceutical product and with three different sensors. The coefficients of variation of the calculated recoveries are included in Table 9.

The coefficient of variation was calculated from three measurements with three PPy/FeCN-SPCE sensors prepared in the same conditions.

The recoveries for L-Tyr for the PPy/FeCN sensor obtained by using the standard addition method were in the range 99.92–103.97%, which clearly demonstrates the applicability in practice of the developed sensor.

These results demonstrate the efficiency of the electrochemical method based on sensors upon L-Tyr quantification.

## 4. Conclusions

The PPy films with the ion doping FeCN, NP, and SDS were successfully synthesized through the CA method and deposited on screen-printed carbon electrodes. The sensors were used in order to detect L-Tyr in standard solutions and pharmaceutical products, and it showed that PPy/FeCN-SPCE had the best electroanalytical performances. Good analytical performances were achieved by using CV, which indicates that this sensor can be used in screening analysis. The method based on using PPy/FeCN-SPCE for the quantification of L-Tyr was verified by the standard addition method, which obtained good recovery values. The electroanalytical method has some important advantages when it comes to laboratory practice such as its precision, reliability, simplicity, and low cost. The sensor exhibits fast response, good sensitivity, and stability for the voltamperometric detection of L-tyrosine, also being useful for the selective determination of complex samples containing different AAs. The voltamperometric method can be used when performing quality control on pharmaceutical products and phytoproducts as well as other samples of interest.

## Figures and Tables

**Table 1:** The thickness of the polymer films.

Electrode	Thickness (µm)
PPy/FeCN-SPCE ^1^	0.163
PPy/NP–SPCE ^2^	0.065
PPy/SDS–SPCE ^3^	0.077

^1^ PPy /FeCN-SPCE—polypyrrole/potassium hexacyanoferrate (II)-screen-printed carbon electrode; ^2^ PPy/NP-SPCE—polypyrrole/sodium nitroprusside-screen-printed carbon electrode; ^3^ PPy/SDS-SPCE—polypyrrole/ sodium dodecyl sulfate-screen-printed carbon electrode.

**Table 2:** The potential and intensity of peaks of PPy modified sensors and immersed in a 0.1 M KCl solution at scan rate of 0.1 V × s^−1^.

Sensor		Electrochemical Parameters					
		E_pa_^1^ (V)	E_pc_^2^ (V)	ΔE ^3^ (V)	I_pa_^4^ (mA)	I_pc_^5^ (mA)	I_pc_/I_pa_
PPy/FeCN-SPCE	Redox system I	−0.32	−0.74	0.42	0.00656	−0.02830	4.31
	Redox system II	0.20	0.03	0.17	0.007264	−0.004976	0.69
PPy/NP-SPCE		−0.06	−0.02	−0.04	0.30196	−0.23940	0.79
PPy/SDS-SPCE		−0.26	−0.09	−0.17	0.39094	−0.25168	0.64

^1^ Potential of the anodic peak; ^2^ Potential of the cathodic peak; ^3^ ∆E = E_pa_ − E_pc_; ^4^ Current of the anodic peak; ^5^ Current of the cathodic peak.

**Table 3:** The potential and intensity of peaks of PPy modified sensors immersed in a 0.1 M KCl and 10^−3^ M L-Tyr solution at a scan rate of 0.1 V × s^−1^.

Sensor		Electrochemical Parameters					
		E_pa_ (V)	E_pc_ (V)	ΔE (V)	I_pa_ (mA)	I_pc_ (mA)	I_pc_/I_pa_
PPy/FeCN-SPCE	Redox system I	−0.28	−0.66	−0.38	0.125608	−0.13802	1.09
	Redox system II	0.19	0.08	0.11	0.094318	−0.085659	0.90
PPy/NP-SPCE		−0.17	−0.12	−0.05	0.376208	−0.317410	0.84
PPy/SDS-SPCE		−0.23	−0.13	−0.10	0.384051	−0.265026	0.68

**Table 4:** Active area surface and roughness factor for the electrodes used in the analysis before and after modification.

Electrode	Solution	Slope (mA·s^1/2^·V^−1/2^)	R^2^	Active Area (cm^2^)	Roughness Factor
SPCE		0.00005820	0.9946	0.0803	0.63
PPy/FeCN-SPCE	0.1 M KCl and 10^−3^ M L-Tyr	0.00085700	0.9972	1.1824	9.41
PPy/NP-SPCE		0.00027890	0.972	0.3847	3.06
PPy/SDS-SPCE		0.00034100	0.9910	0.4700	3.74

**Table 5:** Data achieved from the calibrations curves for PPy/FeCN, PPy/NP, and PPy/SDS sensors at Tyr detection.

Sensor	LOD ^1^ (M)	LOQ ^2^ (M)
PPy/FeCN-SPCE	8.20 × 10^−8^	2.73 × 10^−7^
PPy/NP-SPCE	4.30 × 10^−7^	1.43 × 10^−6^
PPy/SDS-SPCE	3.51 × 10^−7^	1.17 × 10^−6^

^1^ Limit of detection; ^2^ Limit of quantification.

**Table 6:** Performance characteristics of several electrochemical sensors in the detection of tyrosine.

No.	Sensitive Material	Electroanalytical Technique	Linearity Range (M)	LOD (M)	Real Samples Analyzed	Reference
1	In situ copper oxide modified MIPPy coated GCE	DPV ^1^	1.0 × 10^−8^–8.0 × 10^−6^	4.0 × 10^−9^	human serum	[56]
2	MWCNTs-doped Poly(glycine)/Poly(acrylic acid)/SPE	LSV ^2^	4.0 × 10^−7^−1.5 × 10^−4^	1.3 × 10^−7^	human serum	[57]
3	AuNPs/poly (trisamine)/GCE	DPV, EIS ^3^	3.9 × 10^−6^–6.18 × 10^−5^	9.0 × 10^−7^	human blood serum	[58]
4	Poly threonine modified graphite-carbon nanotube paste electrode	CV ^4^	2.0 × 10^–6^–2.5 × 10^–5^	2.9 × 10^–7^	black tea	[59]
5	Poly-L-serine/GCE	Amperometry	3.0 × 10^−7^–1.0 × 10^−4^	1.0 × 10^–7^	commercial amino acid oral solution	[60]
6	MIP-polypyrrole/AuE	SWV ^5^	5.0 × 10^−9^–2.5 × 10^−8^	2.5 × 10^−9^	human plasma	[61]
7	GC/CNT/PEDOT/NF/Crown (glassy carbon/multi-walled carbon nanotubes/poly (3-4-ethylene dioxythiophene/Nafion/Crown)	DPV	6.0 × 10^−8^–2.0 × 10^−5^	4.29 × 10^−10^	blood serum	[62]
8	over-oxidized polypyrrole film on indium tin oxide	CV	1.0 × 10^−13^–1.0 × 10^−6^	1.73 × 10^−12^	-	[63]
9	MIP/pTH/Au@ZIF-67 (molecularly imprinted polyaniline/polythionine/gold nanoparticles@zeolitic imidazolate framework-67 composite ()	DPV	1.0 ×10^−8^–4.0 ×10^−6^	7.9 × 10^−10^	human serum	[64]
10	EB-Ppy-BSA/GCE (Electron beam irradiated polypyrrole nanospheres embedded over bovine serum albumin/)	SWV	1.0 × 10^−7^–8.0 × 10^−4^	8.8 × 10^−9^	black tea and chicken extract	[65]
11	BuCh/GCE (butyrylcholine/glassy carbon electrode)	DPV	4.0 × 10^−6^–1.0 × 10^−4^	4.0 × 10^−7^	mixture of amino acids	[32]
12	cAuNPs/2-AETGO/GCE (cubic gold nanoparticles/2-aminoethanethiol functionalized graphene oxide/glassy carbon electrode)	DPV	1.0 × 10^−9^–2.0 × 10^−8^	1.5 × 10^−10^	milk	[33]
13	GC/RGO/ILC/CNT/Fe–Zn (glassy carbon electrode/graphene oxide/ionic liquid crystal/multi-walled carbon nanotubes/Fe–Zn nanoalloy)	DPV	2.0 × 10^−8^ −5.0 × 10^−5^	5.1 × 10^−9^	biological fluids	[19]

^1^ DPV—Differential pulse voltammetry; ^2^ LSV—Linear sweep voltammetry, ^3^ EIS—Electrochemical impedance spectroscopy, ^4^ CV—Cyclic voltammetry, ^5^ SWV—Square wave voltammetry.

**Table 7:** Precision, reproducibility, and stability of the PPy/FeCN-SPCE sensor.

Precision (RSD%)		Reproducibility (RSD%)	Stability	
Intra-day	Inter-day	3.3	Time (days)	Relative Current response (%)
4.4	5.7		5	97
			16	94
			21	91

**Table 8:** The interference of several amino acids on the quantification of 4 × 10^−6^ M L-Tyr.

Amino Acid	c/M	[Tyr]/M	RE (%)	Recovery (%)
L-Tryptophan	0.001	3.812 × 10^−6^	−4.7	95.3
L-Cysteine	0.001	3.815 × 10^−6^	−4.625	95.375
L-Phenylalanine	0.001	4.813 × 10^−6^	4.575	104.575

c—Concentration of interfering AAs; [Tyr]—Concentration of tyrosine found in the analyzed solution; RE—Relative error in quantification of Tyr.

**Table 9:** The quantitative results obtained for L-Tyr detection in pharmaceuticals by the standard addition method.

Pharmaceutical Product	[Tyr]/M Added	I vs. [Tyr] Equation	[Tyr]/M Found	Recovery (%)
Cebrium EVER NEURO PHARMA [Tyr] 3 × 10^−6^ M	2 × 10^−6^	I = 1.0814c + 5 × 10^−5^ R^2^ = 0.9982	5.17847 × 10^−6^	103.57 ± 1.50
	3 × 10^−6^		6.14944 × 10^−6^	102.49 ± 1.47
	4 × 10^−6^		7.02793 × 10^−6^	100.40 ± 1.08
L-Tyrosine 500 SOLARAY [Tyr] 3 × 10^−6^ M	2 × 10^−6^	I = 1.0869c + 5 × 10^−5^ R^2^ = 0.9975	5.19827 × 10^−6^	103.97 ± 1.55
	3 × 10^−6^		6.15512 × 10^−6^	102.59 ± 1.52
	4 × 10^−6^		7.01997 × 10^−6^	100.29 ± 1.02
Tiroidin PARAPHARM [Tyr] 3 × 10^−6^ M	2 × 10^−6^	I = 1.0937c + 5 × 10^−5^ R^2^ = 0.9991	5.10195 × 10^−6^	102.04 ± 1.53
	3 × 10^−6^		6.0437 × 10^−6^	100.73 ± 1.12
	4 × 10^−6^		6.99461 × 10^−6^	99.92 ± 1.09

## Data Availability

The authors confirm that the data supporting the findings of this study are available within the article.
